# A recurrent mutation in *CRYGD* is associated with autosomal dominant congenital coralliform cataract in two unrelated Chinese families

**Published:** 2011-04-28

**Authors:** Guoxing Yang, Chunlei Xiong, Shanlan Li, Yuanyuan Wang, Jialiang Zhao

**Affiliations:** 1 Department of Opthalmology, Peking Union Medical College Hospital, Chinese Academy of Medical Sciences & Peking Union Medical College, Beijing, China; 2 Department of Opthalmology, Hai Cheng Rehabilitation Hospital, Liaoning, China

## Abstract

**Purpose:**

Congenital cataract is a clinically and genetically heterogeneous lens disorder. The purpose of this study was to identify the mutation responsible for autosomal dominant congenital coralliform cataracts in two Chinese families and to investigate the relationship between virulence genes and lens morphology.

**Methods:**

Patients received a physical examination, and blood samples were collected for DNA extraction. Mutation analysis was performed by direct sequencing of the candidate genes: gammaC-crystallin (*CRYGC*), gammaD-crystallin (*CRYGD*), gammaS-crystallin (*CRYGS*), gap-junction protein, alpha 8 (*GJA8*), gap-junction protein, alpha 3 (*GJA3*), and alphaA-crystallin (*CRYAA*).

**Results:**

The affected individuals in two families had congenital coralliform cataracts. Mutational analysis of the *CRYGD* identified a C→A transversion at nucleotide position c.70 in exon 2, which resulted in a threonine substitution for proline at amino-acid residue 24 (P24T). This mutation was identified in all affected individuals but was not found in healthy relatives or 100 control chromosomes from the same ethnic background.

**Conclusions:**

Our results indicated that the P24T mutation of *CRYGD* was responsible for two Chinese pedigrees with congenital coralliform cataracts. *CRYGD* and coralliform cataracts are highly related, and P24T may be a hot-point mutation for this disorder.

## Introduction

Congenital cataract is a significant cause of vision loss, resulting in approximately one third of all cases of blindness in infants. Congenital cataracts occur in approximately 1–6/10,000 of live births, and one quarter of congenital cataracts are hereditary [1-4]. Hereditary (i.e., Mendelian) cataracts are mostly inherited as autosomal dominant forms, but X-linked and autosomal recessive forms are also observed. Congenital cataracts are a clinically and genetically heterogeneous lens disorder. Phenotypically identical cataracts can result from mutations at different genetic loci and can have different inheritance patterns. While in the same genetic locus or a single large family, phenotypically different cataract types can also be found. To date, about forty genetic loci have been linked to congenital cataracts, and 26 genes have been cloned [5], including crystallins, connexins, heat shock transcription factor-4, aquaporin-0, and beaded filament structural protein-2. The types of mutations and the morphology of the cataracts are considered related [5]. In this study, we report on two congenital coralliform cataract pedigrees caused by the P24T mutation of the gammaD-crystallin (*CRYGD*) gene.

## Methods

### Patients and clinical data

**Figure 1:**
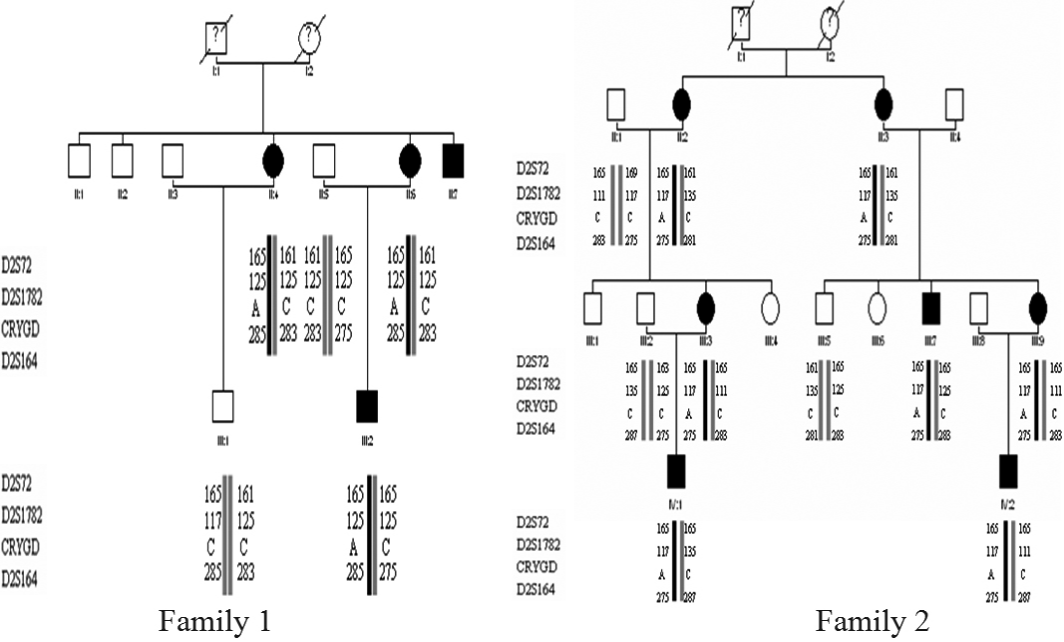
Pedigree and haplotype. Haplotype analysis of the families demonstrated segregation of three microsatellite markers flanking *CRYGD* on chromosome 2q33-q35 region.

Family 1 enrolled in this study was from the Inner Mongolia Autonomous Region, China. The patients had completed a cataract operation, but their vision recovery was not optimal. Family 2 was from Liaoning province, China. These patients had, in part, received cataract operations of one eye; their vision recovery was less than satisfactory. Clinical examination, peripheral blood collection, and DNA extraction were performed at the Department of Ophthalmology, Peking Union Medical College Hospital. Informed consent in accordance with the Declaration of Helsinki and the Institutional Review Board and Ethics Committee of Peking City was obtained from all participants. Family 1 included four confirmed patients, and Family 2 included seven confirmed patients (Figure 1). Clinical data for these subjects was ascertained by detailed ocular examinations.

### Mutation analysis and haplotype analysis

The reported genes, gammaC-crystallin (*CRYGC*), CRYGD, gammaS-crystallin (*CRYGS*), gap-junction protein, alpha 8 (*GJA8*), gap-junction protein, alpha 3 (*GJA3*), and alphaA-crystallin (*CRYAA*) were selected as candidate genes. All coding exons for these genes were amplified by polymerase chain reaction (PCR) using a set of previously described paired primers [6] and presented in Table 1. The PCR products were sequenced using an ABI3730 Automated Sequencer (PE Biosystems, Foster City, CA). Microsatellite markers close to *CRYGD* loci were selected. PCR products from each DNA sample were separated using an ABI 3730XL DNA Analyzer (PE Biosystems).

**Table 1:** Primers used for candidate gene amplification related with congenital cataract.

**Exon**	**primer (5′-3′)**	**Product length (bp)**	**Annealing temperature (°C)**
CRYGD-1–2F	AGAACACGAAAATGCCCTTG		
CRYGD-1–2R	TGCTTGAAACCATCCAGTGA	579	60
CRYGD-3F	GCTGGACTGCCTAACAATGC		
CRYGD-3R	CACATCTTGGTTGCCATTTG	498	58
CRYGS-1F	TTGACTGAAACCAGCCCATA		
CRYGS-1R	TTAGGTGAAAAGCGGGTAGG	166	58
CRYGS-2F	AATTAAGCCACCCAGCTCCT		
CRYGS-2R	AAGCAAGAGAAAGCGGACAG	356	58
CRYGS-3F	CCTGCTGGTGATTTCCATAA		
CRYGS-3R	GATGATGCCTATTTGGACCAC	399	58
GJA8–1F	CGGGGCCTTCTTTGTTCTCTAGTCC		
GJA8–1R	AGGCCCAGGTGGCCCAACTCC	750	67
GJA8–2F	CAGCCGGTGGCCCTGCC		
GJA8–2R	GTTGCCTGGAGTGCACTGCCC	770	66
GJA3–1F	CGGTGTTCATGAGCATTTTC		
GJA3–1R	GACGTAGGTCCGCAGCAG	496	58
GJA3–2F	GCAGGACAATCCCTCGTC		
GJA3–2R	GGTCAGGGCTAGCAGTTTGA	532	58
GJA3–3F	TCGGGTTCCCACCCTACTAT		
GJA3–3R	TGCACTTTGGTTTTGGTTTC	579	58
CRYAA-1 F	CACGCCTTTCCAGAGAAATC		
CRYAA-1 R	CTCTGCAAGGGGATGAAGTG	466	58
CRYAA-2 F	CTTGGTGTGTGGGAGAAGAGG		
CRYAA-2R	TCCCTCTCCCAGGGTTGAAG	377	58
CRYAA-3 F	CCCCCTTCTGCAGTCAGT		
CRYAA-3R	GCTTGAGCTCAGGAGAAGGA	989	58
CRYGC-1–2F	GCAGTATGTACAGGACAGCGTTA		
CRYGC-1–2R	CCTCCCTGTAACCCACATTG	644	58
CRYGC-3F	ATTCCATGCCACAACCTACC		
CRYGC-3R	CCCACCCCATTCACTTCTTA	527	58

## Results

### Clinical findings

**Figure 2:**
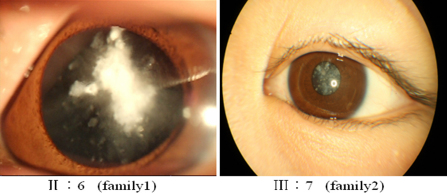
Slit lamp photograph showing coralliform cataract of patient: II:6 in family 1 and III:7 in family 2 (from Figure 1).

All patients enrolled in this study were afflicted with coralliform congenital cataracts (Figure 2), and none of the participants had any other clinical related ophthalmic syndromes.

### Mutation analysis and haplotype analysis

**Figure 3:**
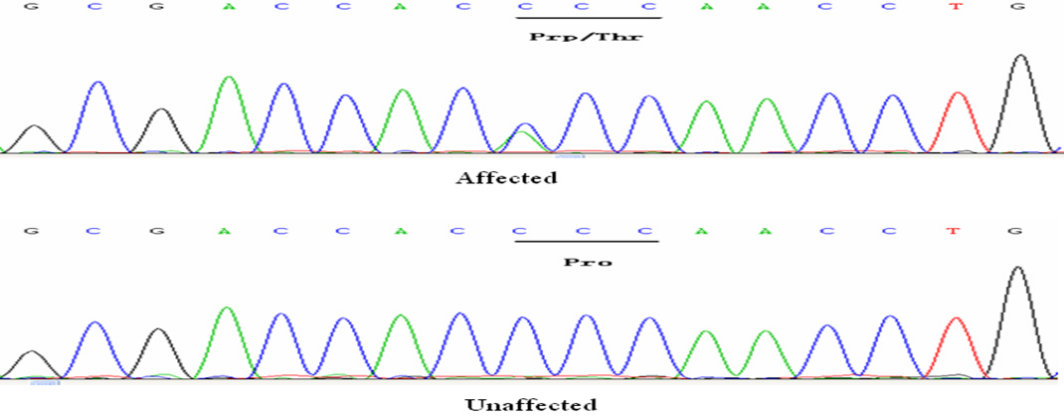
DNA sequences of *CRYGD* in unaffected and affected individuals. The C→A transversion resulted in a threonine substitution for proline at amino-acid residue 24 (P24T) in the affected individuals.

By directly sequencing the coding region of *CRYGD*, we identified a C→A transversion at nucleotide position c.70 in exon 2 of *CRYGD*. This mutation resulted in a threonine substitution for proline at amino-acid residue 24 (P24T; Figure 3). This mutation was only identified in the patients and was not found in healthy relatives or the 100 control chromosomes from the same ethnic background. The disease haplotype cosegregated in all affected members in each family. The haplotype indicated that the two families did not share the same microsatellite markers group. This confirmed that these two families were unrelated (Figure 1).

## Discussion

Coralliform cataract is a special form of congenital cataracts. Several studies have shown that mutations in the *CRYGD* gene, located at 2q33–35, can result in congenital coralliform cataracts, and the P24T mutation of *CRYGD* has been reported in multiple cases. In the two autosomal dominant congenital coralliform cataract pedigrees in this study, we identified a recurrent P24T mutation. This mutation has been found in ten pedigrees, including two cerulean, one lamellar, six coralliform (including our two pedigrees and an unreported pedigree from Tianjin Eye Hospital, China), and one fasciculiform phenotype. From the reported pedigrees, the congenital coralliform cataracts all resulted from *CRYGD* mutations. This information indicated that the coralliform phenotype and the *CRYGD* gene are closely related. Our results supported the idea that virulence genes and lens morphology are related [7-12].

Results of biophysical analysis have shown that the P24T mutant protein has a significantly lower solubility compared with wild-type human γD crystallin. With synchrotron radiation circular dichroism spectroscopy, Evans et al. [13] found that the P24T mutant has a slightly increased content of beta-sheets, due to the substitution of the Pro24 residue, which may be attributed to the extension of an edge beta-strand. This indicates that the insolubility of the P24T mutant protein, rather than the loss of stability, likely causes the occurrences of congenital cataracts. Based on nuclear magnetic resonance analysis, jung et al. found that the pivotal local conformation and dynamics of the threonine substitution in the P24T mutant are different from that of wild-type γD crystallin [14]. The substitution alters motional behavior of the associated region of the protein, speculating that the P24T substitution may initiate aggregation or polymerization. Such aggregation could result in reduced solubility and formation of high-molecular weight complexes.

Up to now, fourteen mutations in *CRYGD* have been reported [15-19]. Several studies have verified that mutation of *CRYGD* can lead to a decrease in solubility of the mutant protein compared to wild type. However, the conformation and stability of the mutant protein undergoes little change.

In conclusion, mutations in *CRYGD* are responsible for coralliform cataracts, and the P24T mutation may be a hot-point mutation affecting the formation of congenital coralliform cataracts.

## References

[1] Rahi JS, Dezateux C (2001). British Congenital Cataract Interest Group. Measuring and interpreting the incidence of congenital ocular anomalies: lessons from a national study of congenital cataract in the UK.. Invest Ophthalmol Vis Sci.

[2] Wirth MG, Russell-Eggitt IM, Craig JE, Elder JE, Mackey DA (2002). Aetiology of congenital and paediatric cataract in an Australian population.. Br J Ophthalmol.

[3] Lambert SR, Drack AV (1996). Infantile cataracts.. Surv Ophthalmol.

[4] Rahi JS, Dezateux C (2000). Congenital and infantile cataract in the United Kingdom: underlying or associated factors.. Invest Ophthalmol Vis Sci.

[5] Hejtmancik JF (2008). Cataracts and their molecular genetics.. Semin Cell Dev Biol.

[6] Santana A, Waiswol M, Arcieri ES, Cabral de Vasconcellos JP, Barbosa de Melo M (2009). Mutation analysis of *CRYAA, CRYGC*, and *CRYGD* associated with autosomal dominant congenital cataract in Brazilian families.. Mol Vis.

[7] Mackay DS, Andley UP, Shiels A (2004). A missense mutation in the gammaD crystallin gene (CRYGD) associated with autosomal dominant “coral-like” cataract linked to chromosome 2q.. Mol Vis.

[8] Shentu X, Yao K, Xu W, Zheng S, Hu S, Gong X (2004). Special fasciculiform cataract caused by a mutation in the gammaDcrystallin gene.. Mol Vis.

[9] Xu WZ, Zheng S, Xu SJ, Huang W, Yao K, Zhang SZ (2004). Autosomal dominant coralliform cataract related to a missense mutation of the gammaD-crystallin gene.. Chin Med J (Engl).

[10] Nandrot E, Slingsby C, Basak A, Cherif-Chefchaouni M, Benazzouz B, Hajaji Y, Boutayeb S, Gribouval O, Arbogast L, Berraho A, Abitbol M, Hilal L (2003). Gamma-D crystallin gene (CRYGD) mutation causes autosomal dominant congenital cerulean cataracts.. J Med Genet.

[11] Khan AO, Aldahmesh MA, Ghadhfan FE (2009). Founder heterozygous P23T CRYGD mutation associated with cerulean (and coralliform) cataract in 2 Saudi families.. Mol Vis.

[12] Santhiya ST, Shyam Manohar M, Rawlley D, Vijayalakshmi P, Namperumalsamy P, Gopinath PM, Löster J, Graw J (2002). Novel mutations in the gamma-crystallin genes cause autosomal dominant congenital cataracts.. J Med Genet.

[13] Evans P, Wyatt K, Wistow GJ, Bateman OA, Wallace BA, Slingsby C (2004). The P23T cataract mutation causes loss of solubility of folded gammaD-crystallin.. J Mol Biol.

[14] Jung J, Byeon IJ, Wang Y, King J, Gronenborn AM (2009). The structure of the cataract-causing P23T mutant of human gammaD-crystallin exhibits distinctive local conformational and dynamic changes.. Biochemistry.

[15] Stephan DA, Gillanders E, Vanderveen D, Freas-Lutz D, Wistow G, Baxevanis AD, Robbins CM, VanAuken A, Quesenberry MI, Bailey-Wilson J, Juo SH, Trent JM, Smith L, Brownstein MJ (1999). Progressive juvenile-onset punctate cataracts caused by mutation of the gammaD-crystallin gene.. Proc Natl Acad Sci USA.

[16] Gu JZ, Qi YH, Lin H, Li X, Wang J, Meng WM, Su H (2006). Autosomal dominant congenital golden crystal nuclear cataract caused by a missense mutation in gammaD crystalline gene (CRYGD) in a Chinese family.. Zhonghua Yan Ke Za Zhi.

[17] Zenteno JC, Morales ME, Moran-Barroso V, Sanchez-Navarro A (2005). CRYGD gene analysis in a family with autosomal dominant congenital cataract: evidence for molecular homogeneity and intrafamilial clinical heterogeneity in aculeiform cataract.. Mol Vis.

[18] Li F, Wang S, Gao C, Liu S, Zhao B, Zhang M, Huang S, Zhu S, Ma X (2008). Mutation G61C in the CRYGD gene causing autosomal dominant congenital coralliform cataracts.. Mol Vis.

[19] Zhang LY, Yam GH, Fan DS, Tam PO, Lam DS, Pang CP (2007). A novel deletion variant of gammaD-crystallin responsible for congenital nuclear cataract.. Mol Vis.

